# The enigma of value: in search of affordable and accessible health care

**DOI:** 10.1007/s10198-016-0857-3

**Published:** 2016-12-02

**Authors:** Thomas D. Szucs, Martina Weiss, Guido Klaus

**Affiliations:** 1 European Center of Pharmaceutical Medicine, University of Basel, Klingelbergstrasse 61, 4056 Basel, Switzerland; 2 Helsana Insurance Group, Postfach, 8081 Zurich, Switzerland

**Keywords:** Pricing, Reimbursement, Value, Hepatitis C, NHS

## Abstract

In times of shrinking resources and pharmaceutical breakthrough situations, our value-assessing systems are stretched to their very limits. Assessing value is highly complex. Current value-assessment systems risk neglecting important factors, such as therapy duration, budget impact, or the importance of combination therapies. Especially when dealing with breakthrough therapies within high-prevalence indications, these factors play an important role in health care spending. When it comes to assessing value in Switzerland, the system is innovation and access-friendly; the price level of pharmaceutical products, however, is relatively high in comparison to neighboring countries. The Swiss pricing and reimbursement system can still improve in terms of efficiency and transparency.

## Introduction

Oscar Wilde once noted: “A cynic is a man who knows the price of everything and the value of nothing.” If value is the ratio between costs and benefits, then Wilde’s “cynic” sees nothing but costs. Further, one could assume that the cynic’s opposite might as well be the “naïve” and sees nothing but benefits. The middle man should theoretically claim value.

A crude translation of Wilde’s saying into the health care setting would give us the image of three protagonists aiming to find the economic equilibrium, “the right price”: the payer (the cynic), the pharmaceutical industry (the naïve one), and the public health authorities (middle man). While the industry keeps vouching for high benefits, payers complain about high costs. The middle men differ from one system to the other. Clearly, what they should bear in mind is preserving solidarity, quality of care, and access within a well-functioning system and promoting innovation at the same time. A tightrope walk, as we will see later on.

The authors aim to show just how imbalanced the price-finding process is and how rough conditions ahead are for two very different systems, given the potentially rising amount of breakthrough pharmaceuticals within widespread indications.

## Market and state failures in Switzerland

Health economists agree: supply and demand cannot explain the dynamics within health care. Supply-induced demand and the asymmetry of knowledge are only two key notions discarding the concept within health care. How do we then define value within health care, more specifically pharmaceuticals?

Whereas many European countries have adopted value-based pricing, Switzerland is legally bound to assess the value of pharmaceutical products solely based on “effectiveness,” “appropriateness,” and “cost-effectiveness.” While Swissmedic, a separate unit, authorizes drugs, the Federal Office of Public Health is responsible for pricing and reimbursement and negotiates prices directly with the pharmaceutical industry. Besides the pharmaceutical companies, no other stakeholders are given access to the negotiations.

Unlike other systems and the subject of public criticism, appraisal and decision are both conducted by the same institution, the Federal Office of Public Health. Each new drug is taken through an assessment that can differ depending on the information available: First, prices are cross-referenced with the prices of nine countries. Second, the new drug can be compared to standard of care therapies and, additionally, can be attributed a price premium for innovation.

Research clearly shows that the Swiss officials could increase cost-effectiveness for pharmaceuticals [1]. However, the authors believe that the recent modifications have not altered the situation. Despite the decreasing gap of pharmaceutical prices between Europe and Switzerland, drug prices in Switzerland remain high. Research shows that greater efficiencies within the pharmaceutical supply chain could be a key to reducing the prices [1]. Despite the very low VAT in comparison to neighboring countries, Switzerland’s drug prices are also driven by the distribution and sales margins of pharmacies, possibly linked to high labor costs in Switzerland [1].

One way or the other, officials are not putting enough effort into bringing the price level down. It is questionable what use external reference pricing is when Switzerland is often among the first to reimburse a pharmaceutical product. It is also questionable whether it makes sense to use foreign list prices which have not been through an HTA process yet. Moreover, most countries are granted important and confidential discounts that Swiss authorities have no knowledge of and are not be taken into account within the reference pricing process.

Another example shows how the method of therapeutic price comparison shows limitations: How do we define the appropriate comparator? Why aren't generic drugs not an appropriate comparator if they are the gold standard? With regards to the increasing number of orphan drugs, set rules are being bypassed.

Various stakeholders criticize the Federal Office for not referring to basic notions of health economy and not applying evidence-based health economic analysis. The Swiss assessors do not asses “cost-effectiveness,” although the legal framework requires the office to do so. One important deficiency within the Swiss pricing and reimbursement system is that de facto authorities do not refer to real-life data despite the fact that this information could be made available. Only real-life data can accurately shed light on actual prescription and administration practices of drugs in comparison to clinical settings.

## Is the grass any greener on the other side?

Given these shortcomings of the Swiss pricing system, it is important to look into other assessment systems and evaluate how the Swiss system can be improved.

Value-based pricing offers a common alternative and is widely used in a number of European countries. However, if we take a closer look, the grass is not any greener on the other side. Countries applying health technology assessment (HTA) show difficulties as well. The assessors often lack important information because of the insufficient quality of evidence when appraising pharmaceutical products [2]. Consequently, less documented products risk being excluded from the markets despite their potentially innovative character, possibly withholding life-saving medicine from the people who need it urgently [2]. It can also be costly to continuously reevaluate a rejected product as soon as new evidence occurs [3]. Moreover, research has shown that countries working with economic evaluations result in diverging recommendations, proving that HTA methods need to be improved [4].

There is another problem with economic evaluations: thresholds. Not only does the question arise where to set a threshold, but once a threshold has been set, how do we deal with incremental cost-effectiveness ratios above that threshold? The UK shows an interesting example: the threshold being set at £20,000 to 30,000 per quality-adjusted life year, many submitted pharmaceuticals are clearly above the threshold and still reimbursed, mostly funded by the Cancer Drug Fund—a separate NHS unit.

Research has found that the National Institute for Health and Care Excellence (NICE) has the highest proportion of submissions above the threshold [5]. Between 2000 and 2014, 34% of all submitted pharmaceuticals were accepted without any restriction and a further 20% with restrictions, much more than HTA systems across Scotland, Australia, and Canada [5]. Mostly a “high clinical benefit over the standard of care” and “addressing an unmet therapeutic need” are reasons for accepting a pharmaceutical despite its over-budget price [5]. One could argue about the use of thresholds when they only apply for every second pharmaceutical submission.

## Sovaldi in Switzerland vs. Sovaldi in the UK: it’s all the same

Let’s look into one of those threshold-surpassing examples: Sovaldi was launched in 2014, a novel therapy within hepatitis C, a disease that was incurable in most cases. Sovaldi definitely changed things for patients suffering from hepatitis C. After treatment with Sovaldi, most patients in fact are cured, unless of course the patient relapses or infects him- or herself with hepatitis C again.

However, Sovaldi has its price. In the US, Sovaldi was also known as the thousand-dollar pill. The media roared. But in fact, Gilead’s plan worked: The incremental cost-effectiveness ratios are favorable for reimbursement because of the high efficacy for patients and savings for future treatments, such as liver transplant, no liver carcinoma treatments and no hospitalization costs.

Nevertheless, Sovaldi caused quite a stir because, but not only because of the high price. Had Sovaldi been a pharmaceutical for a rare disease, reactions would have been different. Sovaldi, however, can potentially be administered to approximately 1% of Western populations. At 1000 dollars per pill, the 84 pills necessary for a 12-week treatment equals a substantial short-term budget impact for any Western health care system, no matter how much money can be saved in the long run. Even though it is safe to say Sovaldi might be cost-effective, this does not mean it is affordable.

In consequence, the Swiss Federal Office of Public Health set limitations on the drug: Sovaldi had only been authorized for patients with advanced hepatitis C, leaving others untreated. The situation slightly improved as competitive drugs entered the market. A 12-week therapy with Sovaldi or Harvoni, however, still costs 50,000 euros combined with ribavirin and interferon.

After the National Institute for Health and Care Excellence (NICE) recommended reimbursement for Sovaldi despite the very high costs per QALY, the NHS introduced Sovaldi, similarly to the Swiss Federal Office of Public Health, with limitations to its use. Pricewise, therapy costs combining Sovaldi with ribavirin and interferon in the UK are not far from the Swiss price, approximately 45,000 euros.

Sovaldi is not an exception. Looking into the future, surely more high-potential pharmaceuticals within wide-spread diseases are ahead. In fact, things could get worse, especially when unlike Sovaldi drugs entail life-long intake on top of other drugs. Promising immunotherapies within oncology or a breakthrough within Alzheimer’s disease will be far more challenging for our systems.

Whether the NHS or the Swiss system, we are failing when it comes to assessing the value of innovative drugs in a widespread disease or with long therapy duration. Sovaldi has shown that economic evaluations, e.g. HTA, manage to justify high prices because of the high benefit, and thresholds manage to crumble. As a result, authorities are forced to differentiate patient groups and limit access. If more such promising drugs are to come and we continue ignoring budget impact, access will become a key issue, driving systems away from solidarity and equity, key components of many health care systems in Western Europe.

## Lessons learned: equilibrium through cooperation

In order to control pharmaceutical spending and increase access for patients, authorities would have to lead more realistic price negotiations with the pharmaceutical industry. Prevalence, therapy duration, and the amount of add-on therapies, all these factors need to be taken into account. The key questions should be: First, what is the benefit of the pharmaceutical? Second, what will this cost be for the treatment of one patient? Third and more importantly, what will the budget impact be, meaning what will the cost be in sum?

As for Switzerland, in terms of *transparency*, the Swiss Federal Office of Public Health should start by including stakeholders in the discussion. The pricing and reimbursement system does not reflect fundamental Swiss political values, such as debate, consensus, and multi-stakeholder agreements. It is not only important to shed more light onto the pricing process, but also to enable stakeholders, such as payers, to participate in the pricing process or at least allow them the right of appeal.

In terms of *efficiency*, a little more HTA couldn’t hurt. What are we paying and what are we getting? Future innovations cannot only be about efficacy and effectiveness: being able to prove a cost-benefit beyond a clinical and in a real-life setting is an essential criterion. They also need to be efficient, meaning value for the money and affordable. A pricing and reimbursement system should be able to simulate the market: greater volume must have an impact on price. The lack of competitiveness and the market failure situation in this particular corner of health care keep prices artificially high.

If health care officials are not able to rethink their pricing and reimbursement systems, the only option left is to reflect on what we can and cannot pay for with public funds.

## Conclusion

If it is in value that we trust, breakthrough situations may lead the way to bankruptcy earlier rather than later in Switzerland and in the UK. Current pricing and reimbursement systems show a lot of “win” for the industry and a fair amount of “lose” for the payer and longer more “lose” for the patient.

Has value become an essential, but not a sufficient component of pricing and reimbursement? Or has our understanding of value become obsolete and needs redefining? Clearly, the Swiss system would not correct its deficits by introducing a value-based pricing system. Introducing economic evaluations would not solve the problem; instead it would add new challenges. Recommendations for the Swiss pricing and reimbursement system are clear: higher efficiency by evaluating value and greater transparency by introducing other stakeholders into the price negotiations.

It seems clear that defining the value of anything within a context of lacking market dynamics is not an easy thing to do and raises ethical, economic, social, and political questions. There is no single formula that we can apply. Other and more differentiating factors need to be considered and require constant revision in the future. Today’s values do not necessarily reflect the market of tomorrow.

Going back to Oscar Wilde’s saying, the authors claim transparency by giving the cynic a voice. Where there is no market, an equilibrium needs to be simulated, especially when a naïve one and middle man are in a “huis-clos” situation. Furthermore, within the Swiss pricing and reimbursement context, the middle man is in a weak negotiation position, lacking important information.

Introducing payers and health economists into the process could surely make health care more affordable in the future, given the important budget impacts ahead. Moreover, authorities need to keep in mind what our systems are based on: solidarity and equity. In order to guarantee a sustainable system, future pharmaceuticals are to be made accessible to patients who are in true need by finding “the right price.”
